# Dual-layer spectral detector CT to study the correlation between pericoronary adipose tissue and coronary artery stenosis

**DOI:** 10.1186/s13019-021-01709-2

**Published:** 2021-11-07

**Authors:** Xiaolong Zhu, Xujiao Chen, Shaowei Ma, Ke Zhou, Yang Hou

**Affiliations:** 1 Department of Radiology, Key Laboratory of Intelligent Computing in Medical Image, Ministry of Education, Shengjing Hospital of China Medical University, No.36, Sanhao Street, Heping District, Shenyang, 110004 Liaoning People’s Republic of China; 2 Department of Medical Imaging, The First Affiliated Hospital of Hebei North University, Zhangjiakou, 075000 People’s Republic of China

**Keywords:** Dual-layer spectral detector, Pericoronary adipose tissue, Epicardial fat volume, Coronary artery stenosis, Plaque

## Abstract

**Background:**

To investigate the relationship of pericoronary adipose tissue (PCAT) with coronary artery stenosis using dual-layer spectral detector CT (SDCT).

**Methods:**

99 patients were retrospectively divided into normal group, non-significant stenosis group and significant stenosis group (n = 33 in each group). Fat attenuation index (FAI) 40kev, spectral curve slope (λHU), effective atomic number (Eff-Z) and epicardial fat volume (EFV) were quantitatively evaluated of the narrowest part of the lesion tissue by SDCT.

**Results:**

There were significant differences in PCAT parameters on SDCT (FAI40keV, λHU, Eff-Z and EFV) among the three groups (*P* < 0.05). FAI40keV, λHU, and Eff-Z in significant stenosis group were statistically different from those in normal group and non-significant stenosis group (*P* < 0.05). FAI40keV, λHU, and Eff-Z in non-significant stenosis group were statistically different from significant stenosis group (*P* < 0.05). EFV in normal group were significantly lower in non-significant stenosis group and significant stenosis group (*P* < 0.001). Univariate and multivariate logistic regression analyses identified FAI40keV (OR = 1.50, 95%CI 1.01 to 1.09) and λHU (OR = 6.81, 95%CI 1.87 to 24.86) as independent predictors of significant stenosis. FAI40keV and λHU had quite good discrimination, with an AUC of 0.84 and 0.80 respectively.

**Conclusion:**

FAI40keV, λHU, and Eff-Z on SDCT in significant stenosis group were significantly different from normal and non-significant stenosis group while EFV in normal group were significantly different from non-significant stenosis group and significant stenosis group. FAI40kev and λHU were risk factors for significant stenosis.

## Background

Coronary artery disease (CAD) is one of the leading causes of death in the world [1]. As well known, the epicardial adipose tissue (EAT) has endocrine functions and shares the microenvironment with the coronary artery. Magnetic resonance imaging (MRI) and multi-slice spiral CT (MSCT) have been used to evaluate EAT, especially the application of MSCT. MSCT quantitative epicardial fat volume (EFV) is an important predictor of coronary heart disease and cardiovascular events, which is also very important for formulating follow-up treatment plans [2, 3].

The EAT surrounding the coronary artery is called pericoronary adipose tissue (PCAT), which has been associated with CAD and major cardiovascular events [4]. Pericoronal fat attenuation index (FAI) is a marker of coronary artery wall inflammation. Antonopoulos et al. [5] proposed that the vessel-derived inflammatory cytokine of PCAT can inhibit the differentiation of human preadipocytes in a paracrine manner and reflect the balance between the lipid and water phase of adipose tissue through FAI, and then reflect the degree of adipocyte differentiation. These findings support the notion that vessel-derived inflammatory cytokines inhibit lipid accumulation in PVAT by inducing the proliferation and inhibiting the differentiation of human preadipocytes in a paracrine manner. Therefore, we hypothesized that noninvasive imaging tools, capable of monitoring these phenotypic changes of PVAT driven by the underlying vascular inflammation, could be used to identify vascular inflammation in human coronary arteries.

Therefore, inflammation can be quantified using FAI. Evaluation of the degree of atherosclerosis and the state of pericoronary fat can help reveal the role of inflammation in the formation and development of atherosclerosis. Although both FAI and EFV are closely related to atherosclerosis, there is no comparison between the two indicators and the degree of lumen stenosis.

Energy CT can identify material components, and the structure of different components can reflect the significant difference in attenuation, atomic number and spectral curve, therefore it may reflect the pathophysiological function of the tissue [6]. Dual-layer spectral detector CT (SDCT) has emerged as a new energy CT with potential to improve the ability to detect tissue characterization. Compared with traditional techniques, effective atomic number based on SDCT can improve the diagnostic performance of coronary artery plaque analysis [7]. In theory, the index of PCAT based on SDCT may be more sensitive than that of MSCT in reflecting the inflammation of PCAT.

This study aims to explore the correlation between PCAT parameters and different degrees of coronary artery stenosis based on SDCT and to provide early warning and early effective treatment for the development of coronary atherosclerosis.

## Materials and methods

### General information

99 patients who underwent coronary computed tomography angiography (CTA) using SDCT for CAD between May 2019 and September 2019 at Shengjing Hospital of China Medical University were retrospectively included. 33 consecutive patients with coronary diameter stenosis (DS) ≥ 50% were retrospectively included and defined as significant stenosis group. 33 patients with coronary DS < 50% and 33 patients without coronary stenosis during the same time period at a ratio of 1:1:1 were included and defined as non-significant stenosis group and control group, respectively.

Inclusion criteria were patients with good CTA image quality (good blood vessel display, clear borders, no respiratory and heartbeat artifacts, no step artifacts). Exclusion criteria were patients (1) with high-risk plaque on CTA; (2) with history of malignancy, acute infection or fever; and (3) with history of cardiac surgery. The study was approved by the ethics committee of Shengjing Hospital of China Medical University (2020PS231K) and all patients signed the written informed consent for examination.

### SDCT acquisition and reconstruction

All patients underwent coronary CTA examinations on a dual-layer SDCT (IQon spectral CT, Philips Healthcare) using prospective electrocardiogram (ECG)-gated acquisitions (Step & Shoot Cardiac). The scan parameters were set as follows: The tube voltage was set at 120 kV; The tube current automatic modulation technology was used, with the Dose Right Index (DRI) of 13; the field of view was 250 mm, the tube rotation time was 0.27 s; the detector collimation was 64 × 0.625 mm, the display matrix was 512 × 512, the rotation speed was 0.27 s, and the detector collimation was 64 × 0.625 mm. The scan trigger was centered around a physiologic cardiac phase of ventricular diastasis corresponding to 78% of the R–R interval with a ± 3% buffer. Automatic bolus tracker was used with a region of interest (ROI) in the ascending aorta at the level of pulmonary artery. The scans were initiated under full inspiration 6 s after a pre-determined signal attenuation threshold of 150 HU were attained. Contrast media (Visipaque Iodixanol 270; GE Healthcare, Ireland) was injected intravenously through the antecubital vein using an 18-gauge catheter dual-tube high pressure syringe (Ulrich REF XD 2051). Contrast media was used individually according to body weight. The total amount of contrast media = patient weight * 0.8 mL/kg. Contrast injection flow rate (mL/s) = total amount (mL)/injection time (12 s), followed by injection of 30 mL of saline at the same injection rate. Prior to CT examination, patients with a heart rate (HR) > 75 bpm received a β-receptor blocker 25–50 mg (Metoprolol Succinate sustained-release tablets, AstraZeneca, Sweden) orally to reduce and stabilize HR.

Spectral reconstruction algorithm (Spectral level 4, Philips Healthcare) were adopted for image reconstruction. The convolution function was CS, with a reconstruction layer thickness of 0.9 mm and interval of 0.45 mm. After the spectral-based image (SBI) were reconstructed, the above image was transmitted to IntelliSpace Protal (Version 6.5, Philips Healthcare) for post-processing.

### Coronary CTA analysis

The coronary artery segment was divided into 17 segments according to the American Heart Association guidelines for coronary artery segmentation [8], and the degree of coronary artery stenosis was measured by image processing software and visual inspection: normal (no stenosis), no significant stenosis (diameter stenosis (DS) < 50%) and significant stenosis(DS ≥ 50%)[9]. Plaques were classified as noncalcified plaques (without any calcium), calcified plaques and mixed plaques (the calcified volume within the plaques ≤ 50%) [10].

### Parameters of EAT quantification

The PCAT parameters around the most significant stenosis were measured and analyzed in significant stenosis group and non-significant stenosis group. In the normal group, PCAT parameters were measured at 40 mm proximal to the right coronary artery (RCA) and the radial distance from the outer vessel wall is equal to the diameter of the target vessel. The whole (average) plaque burden of patients with plaques was 57.47%. The FAI on 40 keV virtual mono-energetic images (VMI) (FAI40keV), the slope of the energy spectrum curve (λHU), and the effective atomic number (Eff-Z) were obtained. FAI was defined as the mean CT attenuation of adipose tissue, which was within a radial distance from the outer vessel wall equal to the diameter of the target vessel [11], and the length covers the 3-dimensional volume of the entire lesion. The attenuation value of PCAT around the narrowest part of the blood vessel with a constant ROI of 20 mm^2^ in the axial position was measured. The composition in the range of polychromatic images − 190 to − 30HU is considered as the fat threshold, and we take an integer as the attenuation threshold on VMI at 40 kev and 70 kev. The relative EAT attenuation levels between VMI at 40 kev and 70 kev levels were 1.44 and 0.90, respectively [12]. Therefore − 280 HU to − 40 HU and − 170 HU to − 30HU were applied to identify adipose tissue voxels in VMI at 40 keV and 70 keV respectively. Measure and calculate PCAT attenuation in VMI at 40 keV (FAI40keV) and subcutaneous fat (SAT), λHU = y/x, y means the difference of absolute value of PCAT attenuation corresponding to 40 keV and 70 keV single level, x value is fixed at 30. Take the average of the three measurements.

**Fig. 1:**
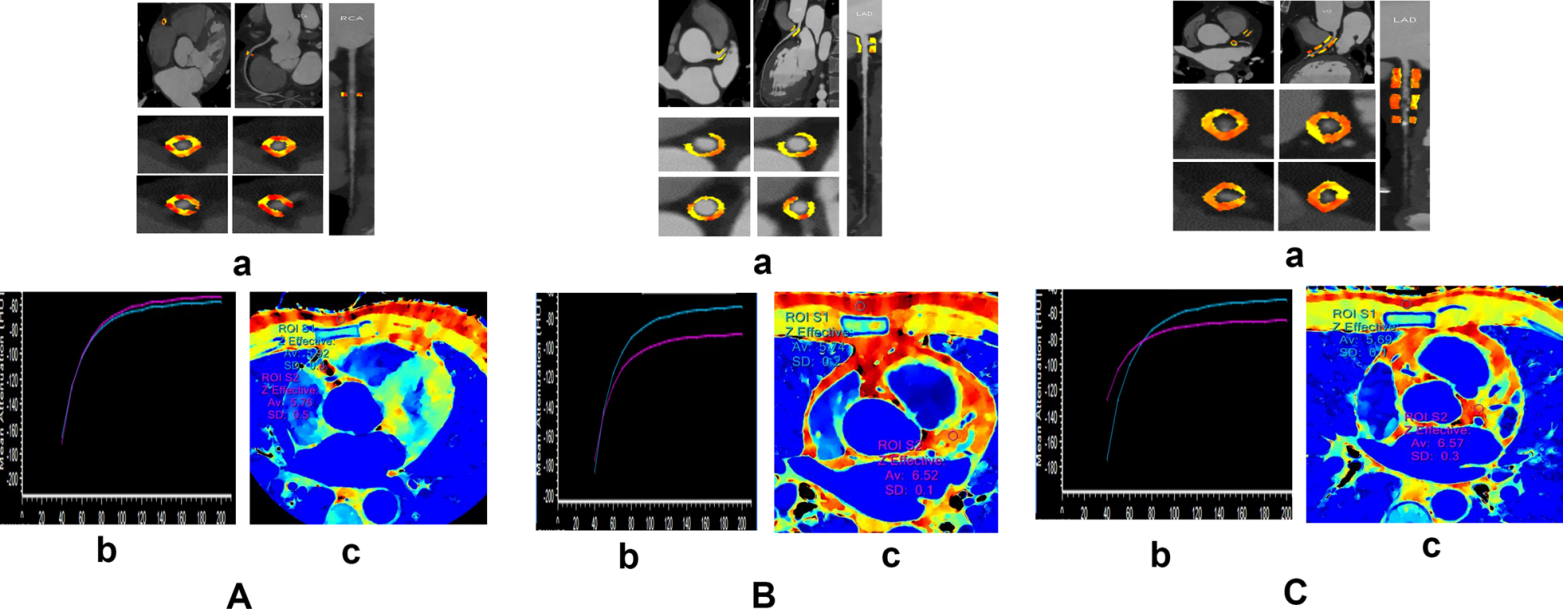
The energy spectrum index of three groups of pericoronary adipose tissue. **A** Normal group. **B** Non-significant stenosis group. **C** Significant stenosis group. a. Measurement of Fat attenuation index (FAI40kev). b. Measurement of spectral curve slope (λHU). c. Measurement of effective atomic number (Eff-Z)

EFV was defined as the volume of all adipose tissue surrounded by pericardium (Fig. 1) and EFV was measured on a conventional polychromatic images. Analysis was independently evaluated by two experienced radiologists (with a 5-year or an 8-year experience of coronary CTA). Take the average of the two as the final measured value. EFV was semi-automatically measured using the software (Heart Disease Risk Assessment Version 1.2.0, Siemens Healthineer, Germany) and manually adjusted if necessary. With the bifurcation of the pulmonary artery as the upper boundary and the level of the descending artery as the lower boundary of the heart, EFV were automatically calculated by including contiguous three-dimensional fat voxels within the range of -190 to -30HU enclosed by the visceral pericardium [13].

### Coronary angiography (CAG)

Patients in significant stenosis group received CAG, and patients in non-significant stenosis group and normal group did not receive CAG. The severity of coronary artery disease was determined by CAG. CAG was performed according to standard techniques and at least two views in different planes were obtained for each coronary artery. The images with the most severe stenosis were captured and the severity of coronary stenosis was evaluated by one experienced observer who was aware of the patients’ clinical history but blinded to the results from coronary CTA evaluated all angiograms with regard to the presence (diameter reduction ≥ 50%).

### Statistical analysis

All data analysis was performed using SPSS. 20.0 (IBM, Armonk, NY) and SAS 9.4 (IBM, Armonk, NY). Quantitative data were described as mean ± standard deviation (SD). Categorical variables were presented as numbers (percentage). Multivariate analysis of variance and Duncan multiple range test were used to compare the basic information of the three groups. Multiple linear regression analysis was performed to evaluate the relationship between FAI40keV, λHU, Eff-Z, EFV and the significant stenosis. Z-test was used to compare the AUC between models. *P* < 0.05 was considered statistically significant.

## Results

### Baseline information

There were no significant difference in age, gender, BMI, hypertension, diabetes, smoking, family history of coronary heart disease, cholesterol, medicine, the narrowest lesion location and the narrowest lesion plaque among the three groups (*P* > 0.05) (Table 1).

**Table 1:** Baseline data of patients

Characteristics	Normal group (n = 33)	Non-significant stenosis group (n = 33)	Significant stenosis group (n = 33)	*P* value
Age	53.12 ± 8.33	55.73 ± 6.80	56.82 ± 9.41	0.297
Gender (Male, n, %)	14(42.42)	21(63.64)	24(72.73)	0.326
BMI	23.78 ± 4.35	25.44 ± 5.12	25.43 ± 6.43	0.124
Hypertension (n, %)	17 (51.52)	21 (63.64)	22 (66.67)	0.215
Diabetes (n, %)	9 (27.27)	13 (39.39)	15 (45.45)	0.413
Smoking (n, %)	11(33.33)	18 (54.55)	17 (51.52)	0.158
Family history of CHD (n, %)	8(24.24)	9(27.27)	13(39.39)	0.594
HDL cholesterol	1.05 ± 0.87	0.99 ± 0.76	0.92 ± 0.81	0.252
LDL cholesterol	2.54 ± 0.67	2.91 ± 0.83	3.26 ± 0.78	0.127
Total cholesterol	4.18 ± 0.48	4.59 ± 0.62	4.91 ± 0.43	0.234
Triglyceride	1.62 ± 0.32	2.08 ± 0.48	2.45 ± 0.51	0.211
*Medicine*				
Aspirin	31(93.94)	33(100)	33(100)	0.761
Statins	22(66.67)	31(93.94)	33(100)	0.436
*The narrowest lesion location*				
LAD (n, %)		24(72.73)	28(84.85)	
RCA (n, %)		7(21.21)	4(12.12)	
LCX (n, %)		1(3.03)	1(3.03)	
LM (n, %)		1(3.03)	0(0)	
*The narrowest lesion plaque*				
Non-calcified		8(24.24)	9(27.20)	
Calcified plaque		11(33.33)	4(12.10)	
Mixed		14(42.40)	20(60.60)	

BMI = body mass index; CHD = coronary heart disease; LM = left main coronary artery; LAD = left anterior descending artery; LCX = left circumflex artery; RCA = right coronary artery, HDL = high-density lipoprotein, LDL = low-density lipoprotein. *P* < 0.05 was considered statistically significant

### Comparison of PCAT parameters on SDCT in three groups

The reproducibility of FAI measurements was good, and the Kappa value of intro-observer agreements was 0.848 (95% CI, 0.748 ~ 0.952, *P* < 0.001). As shown in Table 2, there were significant differences in PCAT parameters on SDCT (FAI40keV, λHU, Eff-Z and EFV) among the three groups (*P* < 0.05). The indexes of PCAT (FAI40keV, λHU, and Eff-Z) in significant stenosis group were statistically different from those in normal group and non-significant stenosis group (*P* < 0.05). The indexes of PCAT (FAI40keV, λHU, and Eff-Z) in non-significant stenosis group were statistically different from those in significant stenosis group (*P* < 0.05). The EFV in normal group were significantly lower than those in non-significant stenosis group and significant stenosis group (*P* < 0.001).

**Table 2:** Quantification of PCAT parameters in three groups

	Normal group (n = 33)	Non-significant stenosis group (n = 33)	Significant stenosis group (n = 33)	F value	*P* value
FAI_40keV_	− 168.73 ± 34.17	− 161.63 ± 36.31^#^	− 127.22 ± 33.28*	24.78	0.003
λ_HU_	1.89 ± 0.92	1.96 ± 1.17^#^	1.18 ± 0.83*	21.73	0.004
Eff -Z	6.15 ± 0.64	6.13 ± 0.56^#^	6.61 ± 0.61*	18.34	0.005
EFV	102.11 ± 35.59^&^	129.17 ± 39.78	140.06 ± 35.50*	20.28	0.004

Compared with non-significant stenosis group, ^&^*P* < 0.05; compared with significant stenosis group, ^#^*P* < 0.05; compared with normal group, *P < 0.05

PCAT = pericoronary adipose tissue

### Relationship between PCAT parameters and significant stenosis

Variables in Table 2 were assessed in univariate and multivariate logistic regression analyses, which identified FAI40keV (OR = 1.50, 95%CI 1.01 to 1.09) and λHU (OR = 6.81, 95%CI 1.87 to 24.86) as independent predictors of significant stenosis (Table 3).

**Table 3:** Odds ratios for significant stenosis (stenosis ≥ 50%)

	Univariate			Multivariate		
	β	OR(95%CI)	*P* value	β	OR(95%CI)	*P* value
FAI40keV	0.041	1.027(1.001–1.054)	0.042	0.037	1.496(1.013–1.087)	0.023
λ_HU_	0.434	2.939(1.283–6.733)	0.011	0.561	6.813(1.868–24.861)	0.014
Eff -Z	1.677	1.167(0.186–6.942)	0.869	0.154	0.142(0.133–1.647)	0.183
EFV	0.032	1.016(0.998–1.035)	0.075	0.016	1.004(0.998–1.009)	0.503

OR = Odds ratio; CI = confidence interval

### ROC analysis

**Fig. 2:**
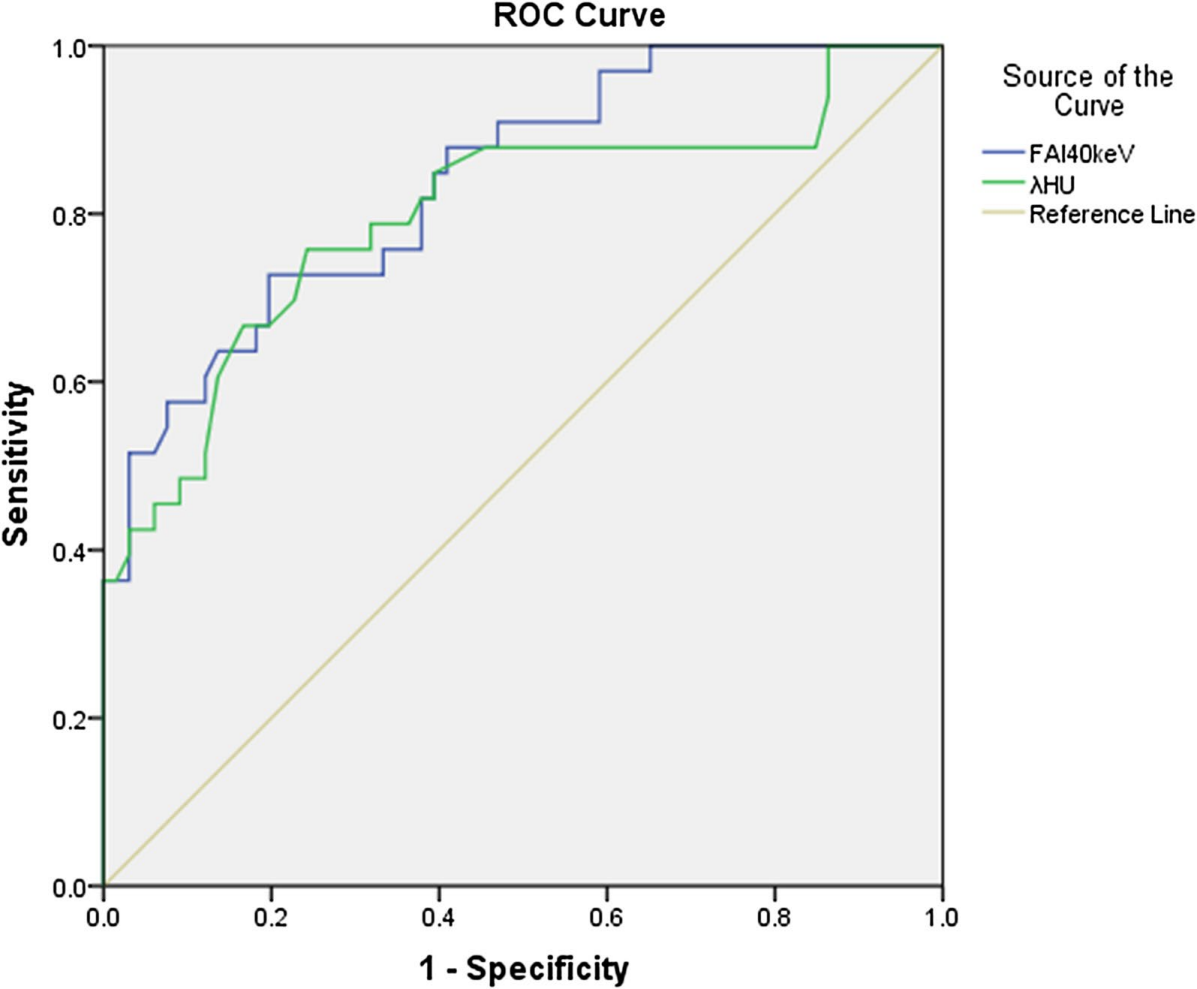
ROC analysis for prediction of significant stenosis

The ROC showed that FAI40keV and λHU had quite good discrimination (Fig. 2), with an AUC of 0.84 and 0.80 respectively (Table 4). No significant differences were observed in FAI40keV and λHU in the prediction for significant stenosis (*P* = 0.936).

**Table 4:** Cut-off values of PCAT indices for the detection of significant stenosis

Parameters	Cut-off value	AUC (95% CI)	Sensitivity (%)	Specificity (%)	Positive likelihood ratio	Negative likelihood ratio	Accuracy (%)
FAI_40keV_	− 144.21	0.84(0.76–0.92)	72.70	80.30	3.69	0.34	81.80
λ_HU_	1.55	0.80(0.70–0.90)	75.80	75.80	3.13	0.32	75.70
Z value		0.081					
*P* value		0.936					

PCAT = pericoronary adipose tissue; CI = confidence interval

## Discussion

In this study, we found that the indexes of PCAT (FAI40keV, λHU, and Eff-Z) on SDCT in significant stenosis group were significantly different from those in normal group and non-significant stenosis group while EFV in normal group were significantly different from those in non-significant stenosis group and significant stenosis group. FAI40keV and λHU are independent predictors of significant stenosis. To the best of our knowledge, this article is the first application of SDCT to study the changes of PCAT parameters at different stages of atherosclerosis.

Vascular inflammation is not only a key factor in the formation of atherosclerotic plaque, but also in triggering the plaque rupture [14, 15]. There is evidence that the metabolic process of PCAT induces endothelial dysfunction, inflammatory response and smooth muscle cell proliferation, thereby affecting the formation of atherosclerotic plaques [16]. EAT is believed to exert local inflammation and atherosclerosis effects on epicardial coronary arteries through a paracrine mechanism, and is therefore related to the pathogenesis of coronary atherosclerosis [17, 18]. All stages of atherosclerosis involve the inflammatory process and were accompanied by multiple inflammatory mediators. Compared with conventional CT, SDCT uses X-ray photons of different energies, which can penetrate different materials and produce differential compton effect and photoelectric effect. Due to the difference of atomic K boundary value, we can get different effective atomic numbers, energy spectrum curves, single-energy images, and can detect differences in constituent substances.

The results of this study showed there were significant differences in SDCT-based PCAT parameters (FAI40keV, λHU, Eff-Z and EFV) among the three groups. The indexes of PCAT (FAI40keV, λHU, and Eff-Z) on SDCT in significant stenosis group were significantly different from those in normal group and non-significant stenosis group while EFV in normal group were significantly different from those in non-significant stenosis group and significant stenosis group. In addition, this study showed that FAI40keV and λHU are independent predictors of significant stenosis. This prompted PCAT attenuation may provide a surrogate global biomarker for underlying vascular inflammation importantly [19]. Some studies have shown that the EFV of patients with cardiovascular disease is related to the severity of the disease [20], and the EFV of patients with coronary heart disease is larger than patients without coronary heart disease [21], however some clinical studies did not find a significant correlation between the EFV and the severity of coronary artery stenosis [22], which is consistent with the results of this present study. This may indicate that in the process from subclinical atherosclerosis to non-significant stenosis, the increase in EFV has a better warning effect on atherosclerosis than the PCAT indicators, and EFV will help identify people at high risk of atherosclerosis. This just makes up for the relatively insensitive shortcoming of PCAT indicators in the early stage of the disease.

The small sample size was a limitation of our study and there is a bias in the selection of retrospective studies. Therefore, a large randomized controlled study remain to be done in the future. We did not include patients with high-risk plaques considering the impact of high-risk plaque on PCAT. In addition, we did not compare the value of FAI on conventional CT and SDCT in the diagnosis of vascular stenosis. We did not measure the effect of different measurement points to our findings.

In conclusion, the PCAT parameters of SDCT changed with the change of stenosis. FAI40kev and λHU were independent risk factors for significant coronary artery stenosis. SDCT may provide alternative imaging markers for potential vascular inflammation, which may help indicate the degree of disease progression.

## Data Availability

Data sharing not applicable to this article as no datasets were generated or analysed during the current study.

## Abbreviations

PCAT: Pericoronary adipose tissue
SDCT: Spectral detector CT
FAI: Fat attenuation index
λHU: Spectral curve slope
Eff-Z: Effective atomic number
CAD: Coronary artery disease
EAT: Epicardial adipose tissue
MRI: Magnetic resonance imaging
MSCT: Multi-slice spiral CT
EFV: Epicardial fat volume
CTA: Computed tomography angiography
ECG: Electrocardiogram
DRI: Dose right index
ROI: Region of interest
HR: Heart rate
SBI: Spectral-based image
DS: Diameter stenosis
VMI: Virtual mono-energetic images
CAG: Coronary angiography
